# Metabolomic Analysis and Mode of Action of Metabolites of Tea Tree Oil Involved in the Suppression of *Botrytis cinerea*

**DOI:** 10.3389/fmicb.2017.01017

**Published:** 2017-06-06

**Authors:** Jiayu Xu, Xingfeng Shao, Yonghua Li, Yingying Wei, Feng Xu, Hongfei Wang

**Affiliations:** Department of Food Science and Engineering, Ningbo UniversityNingbo, China

**Keywords:** tea tree oil, *Botrytis cinerea*, antifungal mechanism, metabolomics, TCA cycle

## Abstract

Tea tree oil (TTO), a volatile essential oil, has been widely used as an antimicrobial agent. However, the mechanism underlying TTO antifungal activity is not fully understood. In this study, a comprehensive metabolomics survey was undertaken to identify changes in metabolite production in *Botrytis cinerea* cells treated with TTO. Significant differences in 91 metabolites were observed, including 8 upregulated and 83 downregulated metabolites in TTO-treated cells. The results indicate that TTO inhibits primary metabolic pathways through the suppression of the tricarboxylic acid (TCA) cycle and fatty acid metabolism. Further experiments show that TTO treatment decreases the activities of key enzymes in the TCA cycle and increases the level of hydrogen peroxide (H_2_O_2_). Membrane damage is also induced by TTO treatment. We hypothesize that the effect of TTO on *B. cinerea* is achieved mainly by disruption of the TCA cycle and fatty acid metabolism, resulting in mitochondrial dysfunction and oxidative stress.

## Introduction

*Botrytis cinerea* is a necrotrophic fungal pathogen that causes gray mold, a seriously destructive postharvest disease that affects a variety of fresh fruits (Saito et al., 2016). Even at low temperature during storage and transportation, it can produce significant degradation in fruit quality, resulting in severe economic losses. Because chemical fungicides are harmful to human health and the environment, there is an urgent need to develop safe and effective antifungal agents to control the decay caused by *B. cinerea*. Natural antimicrobial agents such as essential oils provide one promising alternative. For example, the essential oils of *Origanum compactum* and *Thymus glandulosus* strongly inhibit the growth of mycelia in *B. cinerea* (Bouchra et al., 2003), and essential oils from three *Monarda* species have antifungal effects on both mycelia and spores (Adebayo et al., 2013). *Syzygium aromaticum, Brassica nigra*, and *Solidago canadensis* L. essential oils also exhibit effective antifungal activity in strawberries against *B. cinerea in vitro* and *in vivo* (Aguilar-González et al., 2015; Liu et al., 2016).

Tea tree oil (TTO), the volatile essential oil extracted from leaves and branches of the Australian native plant *Melaleuca alternifolia* by steam distillation, is frequently used as a topical medicinal and antimicrobial agent (Pazyar et al., 2013). Because of its bioactivities, its efficacy has recently been evaluated against *Staphylococcus aureus, Escherichia coli, Aspergillus niger*, and *Candida albicans in vitro* (Gustafson et al., 1998; Hammer et al., 2003; Shi et al., 2016). TTO controls the growth of *B. cinerea* more effectively than pine and cinnamon oil (Szczerbanik et al., 2007), or clove oil and garlic oil (Cheng and Shao, 2011). TTO also effectively inhibits the growth of *B. cinerea* on Dutch White cabbage (Bishop and Reagan, 1998) and exhibits high antifungal activity on *B. cinerea* in strawberry fruit (Shao et al., 2013b).

Essential oils are generally thought to exert their antifungal activity by damaging plasma membranes (Hood et al., 2010; Khan et al., 2010) and mitochondria (Tian et al., 2012; Zheng et al., 2015; Li et al., 2016). Microscopic observations revealed that the essential oils of *Origanum syriacum* L. var. *bevanii* and *Lavandula stoechas* L. var. *stoechas* caused considerable morphological degenerations of the fungal hyphae of *B. cinerea* such as cytoplasmic coagulation, vacuolations, hyphal shriveling and protoplast leakage, and loss of conidiation (Soylu et al., 2010). Our previous studies confirmed that TTO primarily targets the *B. cinerea* cell wall, resulting in increased membrane permeability, the release of cellular material, and eventual cell death (Shao et al., 2013a). Terpinen-4-ol, the major characteristic component of TTO, severely compromises membrane integrity and increases permeability. Another characteristic component in TTO, 1,8-cineole, damages cellular organelles but does not affect membrane permeability (Yu et al., 2015). These findings are important clues for understanding TTO antifungal activity, although the underlying mechanisms are still not fully clear.

Metabolomics is an emerging technology that provides a comprehensive quantitative and qualitative inventory of the low molecular weight metabolites in a cell or organism (Fiehn et al., 2000). It is particularly useful for analyzing changes in the endogenous metabolism of a biological system that has been stimulated or disturbed, and for identifying metabolic pathways. Recently, metabolomics has been widely applied to study the mechanisms by which different agents exert their antimicrobial effects against fungi and bacteria. For example, the activity of amphotericin B against *C. albicans* can be attributed to changes in metabolite production (Cao et al., 2013). Another study found that the microbiostatic effect of ε-Poly-L-lysine on *Saccharomyces cerevisiae* is achieved by breaking the balance of intracellular metabolites due to disruptions in cell membrane function (Bo et al., 2014). Finally, a metabolomics approach was used to demonstrate that cinnamaldehyde changes the metabolism of *E. coli* by interacting with different biochemical targets (Mousavi et al., 2016).

No studies have yet employed metabolomics to examine changes in fungi in the presence of essential oils. In order to examine the antifungal effects of TTO on *B. cinerea* in detail, we conducted a metabolomics analysis using an ultra-high pressure liquid chromatography system coupled to a quadrupole time-of-flight mass spectrometer (UHPLC-Q-TOF MS).

This study was designed to reveal the mechanisms responsible for the antifungal effects and stress caused by TTO in *B. cinerea* by generating a high-resolution metabolic fingerprint and detecting changes in metabolite levels at high sensitivity.

## Materials and methods

### Fungal isolates, essential oil, and reagents

A highly virulent strain of *B. cinerea* was isolated from strawberries and cultured at 25°C on potato dextrose agar medium (PDA; containing 1 L of an infusion from potatoes, 20 g/L glucose, and 15 g/L agar) before use in experiments. TTO was purchased from Fuzhou Merlot Lotus Biological Technology Company (Fujian Province, China). Ammonium acetate and ammonium hydroxide were purchased from Sigma Aldrich (St. Louis, MO, USA). Acetonitrile and methanol were purchased from Merck (Germany). Ammonium acetate and acetonitrile were of HPLC-grade. Distilled water was filtered through a Milli-Q system from EMD Millipore Corporation (Billerica, MA, USA). Succinate dehydrogenase (SDH), malic dehydrogenase (MDH), citrate synthase (CS), isocitrate dehydrogenase (ICDH), α-ketoglutarate dehydrogenase (α-KGDH), and hydrogen peroxide (H_2_O_2_) reagent kits were purchased from Nanjing Jian Cheng Bioengineering Institute (Nanjing, China).

### Fungal culture and sample preparation

*B. cinerea* cultures were maintained on PDA at 25°C for 3 days. The spore suspension was harvested by adding 10 mL sterile 0.9% NaCl solution to each petri dish and gently scraping the mycelial surface three times with a sterile L-shaped spreader to free the spores. The spore suspension was adjusted using a hemocytometer to 5 × 10^6^ spores/mL. One milliliter suspension was inoculated into 250 mL flasks containing 150 mL sterile potato dextrose broth (PDB; containing 1 L of an infusion from potatoes, 20 g/L glucose) medium and cultured at 25°C on a rotary shaker at 150 revolutions per minute (rpm) for 3 days. TTO was added to the medium to a final concentration of 5 mL/L, and cultures incubated for 2 h. Mycelia were then collected, rinsed three times with 0.1 M phosphate buffer saline (PBS; pH = 7.4), and immediately frozen in liquid nitrogen. Samples were stored at −80°C. Cultures without TTO were used as a control. Six samples of each group were prepared in parallel for LC-MS/MS analysis and other tests.

### LC–MS/MS analysis

Before analysis, control and treated mycelia (~100 mg wet weight) were thawed at 4°C and homogenized. One milliliter of acetonitrile and methanol (1:1, v/v) was added to each sample and mixed by vortexing. Samples were immediately frozen in liquid nitrogen, subjected to ultrasonic grinding for 10 min, stirred for 60 min at −20°C, and then separated by centrifugation at 12,000 × g for 15 min at 4°C. An aliquot of 900 μL supernatant was dried under vacuum, dissolved in 100 μL of acetonitrile and water (1:1, v/v) by vortexing, and centrifuged at 12,000 × g for 15 min at 4°C. The chemical compositions of the samples were analyzed using an UHPLC (1290 Infinity LC, Agilent Technologies) equipped with a quadrupole time-of-flight (AB Sciex TripleTOF 6600) mass spectrometer.

For separation by hydrophilic interaction liquid chromatography (HILIC), samples were analyzed using a 2.1 mm × 100 mm ACQUIY UPLC BEH 1.7 μm column (Waters Corporation, Ireland). In both electron spray ionization (ESI) positive and negative modes, the mobile phase contained (A) 25 mM ammonium acetate and 25 mM ammonium hydroxide in water, and (B) acetonitrile. The elution gradient was: 0–1 min, 85% B; 1–12 min, 85–65% B; 12–12.1 min, 65–40% B; 12.1–15 min, 40% B; 15–15.1 min, 40–85% B; 15.1–20 min, 85% B, with a 5 min re-equilibration period employed.

The ESI source conditions were set as follows: Ion Source Gas1 (Gas1) as 60, Ion Source Gas2 (Gas2) as 60, curtain gas (CUR) as 30, source temperature: 600°C, Ion Spray Voltage Floating (ISVF) ±5,500 V. In the MS only acquisition mode, the instrument was set to acquire over the m/z range 60–1,000 Da, and the accumulation time for the TOF MS scan was set at 0.20 s/spectrum. In the auto MS/MS acquisition mode, the instrument was set to acquire over the m/z range 25–1,000 Da, and the accumulation time for product ion scan was set at 0.05 s/spectrum. The product ion scan was acquired using information dependent acquisition (IDA) with the high sensitivity mode selected. The collision energy (CE) was fixed at 35 V ± 15 eV. The declustering potential (DP) was set to ±60 V.

### Determination of effect of TTO on the leakage of cell membrane

In order to assess TTO affect on the leakage of cell membrane, the absorbance at 260 nm of *B. cinerea* cell was determined. As described above for fungal culture and sample preparation, TTO was added to PDB medium at a final concentration of 5 mL/L. PDB without TTO served as the control. Treated and control group were centrifuged at 10,000 × *g* for 10 min at 4°C, and the absorbance of the supernatant was measured immediately at 260 nm with a UV/Vis spectrophotometer (UV-2000, UNICO Instrument Co., Ltd., Shanghai, China). After 2 h incubation with or without TTO, treated and control group were centrifuged and the absorbance at 260 nm of the obtained supernatant were measured. Each treatment was performed in triplicates. The percentage change in A_260nm_ (Liu et al., 2004) was calculated using the following formula:

$${\text{A}}_{260\text{ nm}}\text{ percentage change}(\%)=[({\text{A}}_{\text{t}}-{\text{A}}_{0})/{\text{A}}_{0}]\times 100$$

where A_0_ is the mean A_260nm_ for cultures measured after 0 h of treatment, and A_t_ is the mean A_260nm_ for cultures after 2 h of treatment.

### Activities of key enzymes involved in tricarboxylic acid (TCA) cycle

Control and treated mycelia were washed with 0.1M PBS (pH = 7.4) three times and then ground in liquid nitrogen. The ground material was suspended in 10 mL 0.05M PBS (pH = 7.2) and centrifuged at 10,000 × *g* for 10 min at 4°C. Enzyme activities were measured in the supernatant for SDH, MDH, CS, ICDH, and α-KGDH, using commercially available kits purchased from Nanjing Jiancheng Bioengineering Institute (Nanjing, China) following the manufacturer's instructions. ICDH, MDH, and α-KGDH activities were detected at 340 nm in redox reaction assays. SDH and CS activities were determined at 600 and 412 nm, respectively. All tests were performed in triplicate.

### Determination of effect of TTO on H_2_O_2_ accumulation

Control and treated mycelia (1 g wet weight) were mixed with 5 mL 0.05 M PBS (pH = 7.0, containing 3% polyvinyl pyrrolidone), subjected to ultrasonic grinding for 15 min, and centrifuged at 10,000 × *g* for 10 min at 4°C. The supernatant was used to determine H_2_O_2_ content of the fungal cells using assay kits obtained from Nanjing Jiancheng Institute of Bioengineering (Nanjing, Jiangsu, China) following the manufacturer's instructions. H_2_O_2_ content was expressed as mmol/g prot. All tests were performed in triplicate.

### Data processing

The raw MS data (.wiff scan files) were converted to MzXML format using ProteoWizard MSConvert and processed using XCMS for feature detection, retention time correction, and alignment. Metabolites were identified by matching high accuracy (<25 ppm) MS/MS data to our standards database.

### Statistical analysis

In the extracted ion features, only variables having more than 50% of the non-zero measurement values in at least one group were retained. For multivariate statistical analysis, the Metabo Analyst (http://www.metaboanalyst.ca) web-based system was used. After Pareto scaling, principal component analysis (PCA) and partial least-squares-discriminant analysis (PLS-DA) were performed. Leave-one-out cross-validation and response permutation testing were used to evaluate the robustness of the model. Metabolites exhibiting significant differences were identified based on the combination of a statistically significant threshold of variable influence on projection (VIP) values obtained from a PLS-DA model and a two-tailed Student's *t*-test (*p*-value) on the raw data. Metabolites with VIP-values larger than 1.0 and *p* < 0.05 were considered significant. All enzyme activity and H_2_O_2_ content data were analyzed using SAS software (Version 8.2; SAS Institute, Cary, NC, USA). These data were analyzed by one-way analysis of variance (ANOVA). Comparison of means was performed by Duncan's multiple range tests. A value of *P* < 0.05 was considered statistically significant.

## Results

### Multivariate analysis

**Figure 1:**
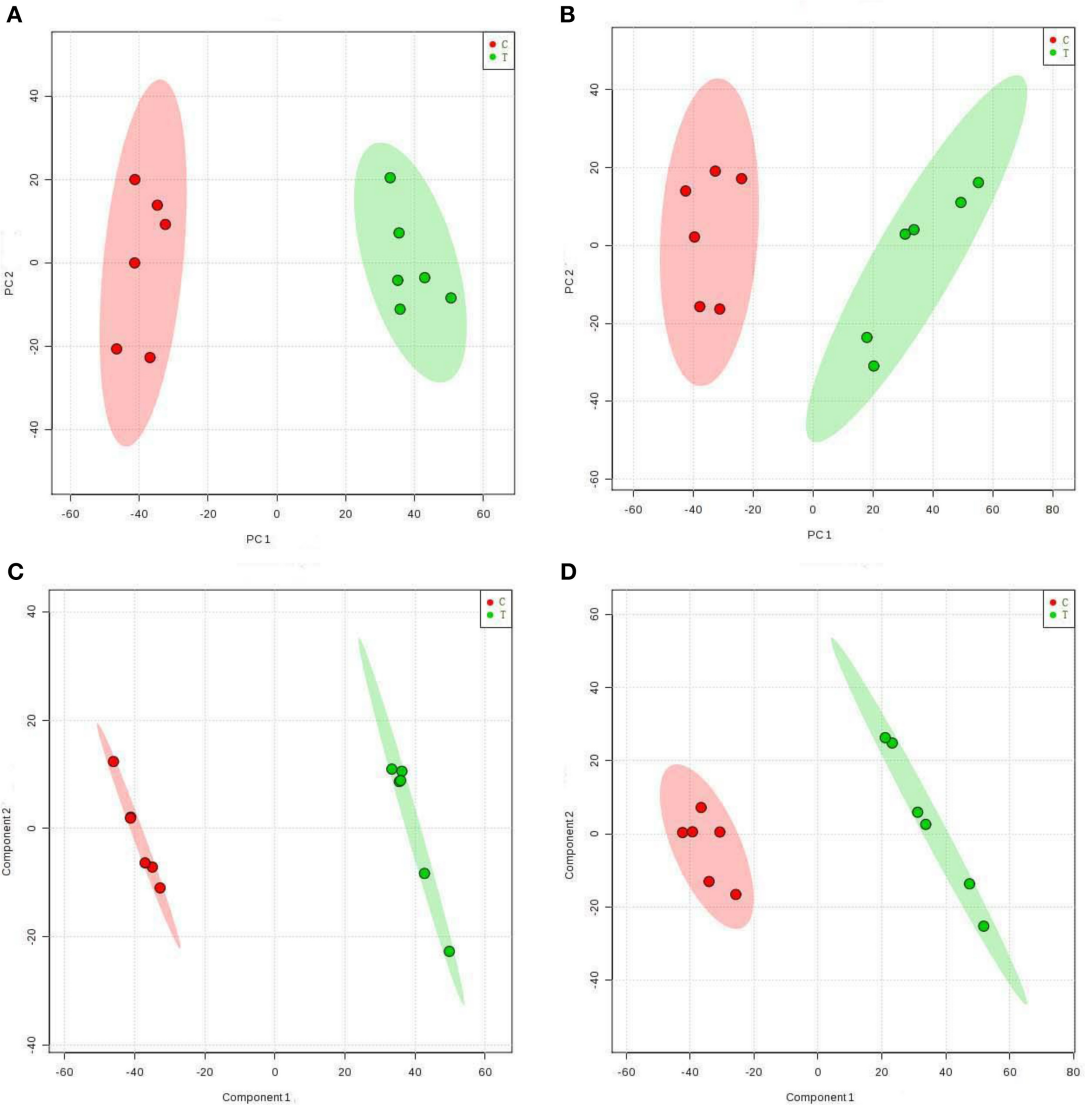
Score plots representing PCA and PLS-DA results based on the UHPLC-Q-TOF MS spectra *B. cinerea* mycelia from the TTO-treated cells (green dots) and control cells (red dots) are shown in **(A,C)** positive and **(B,D)** negative modes. C, control sample; T, TTO-treated sample.

Extracts from TTO-treated and untreated *B. cinerea* were analyzed using a UHPLC-Q-TOF mass spectrometer in positive and negative ion modes. Multivariate analysis was applied to find potential biomarkers. PCA, an unsupervised pattern recognition method, was performed to examine intrinsic variation in the data set. Samples that have similar metabolomics profiles are clustered together and those that are different are placed further apart in PCA score plots. As shown by the PCA score plots in positive (Figure 1A) and negative (Figure 1B) ion modes, the control group and TTO-treated group clustered separately, which indicates that metabolite levels were altered in the TTO-treated group.

To further identify ion peaks that could discriminate between the groups, a supervised pattern recognition method (PLS-DA) was suitable for the selection of biomarkers in similar metabolic profiling data (Schneider et al., 2009). In the PLS-DA score plots for both the positive (Figure 1C) and negative (Figure 1D) ion modes, metabolic differences made it possible to resolve the control and TTO-treated groups into distinct clusters. The *R*^2^ and *Q*^2^ parameters are used to evaluate the quality of the PLS-DA model. The *R*^2^ and *Q*^2^ were 0.99 and 0.97 in positive ion mode, respectively, and *R*^2^ and *Q*^2^ were 0.99 and 0.96 in negative ion mode, indicating that the model had high predictability and reliability.

### Selection and identification of biomarkers

To find the endogenous metabolites that contributed most to the separation of the control and TTO-treated samples, VIP scores were determined after PLS-DA. VIP measures the contribution made by each variable to the PLS-DA model. Ions were considered as potential biomarkers if they satisfied the criteria VIP-value > 1 and *p*-value < 0.05. Based on this algorithm, 91 metabolite ions were identified as primary contributors to the separation between control and TTO-treated groups. The data are summarized in Table 1, which also classifies the metabolites as either upregulated (8 metabolites) or downregulated (83 metabolites).

**Table 1:** Identification and changes in levels of potential biomarkers in *B. cinerea* cells.

**Metabolite**	**Ionization mode**	**RT (min)**	**VIP**	**Fold change**	***P*-value**	**m/z**	**Trend (treated vs. control)**	**Functional category**
Trehalose	ESI(−)	8.68	1.75	0.08	8.53 × 10^−7^	401.12816	↓	Oxidative stress
Succinate	ESI(−)	8.21	1.91	0.05	8.17 × 10^−8^	117.02004	↓	TCA cycle
Myristic acid	ESI(−)	1.18	1.87	0.06	3.89 × 10^−5^	287.22120	↓	Lipid metabolism
L-Malic acid	ESI(−)	11.17	1.41	0.17	2.02 × 10^−5^	133.01430	↓	TCA cycle
L-Glutamate	ESI(+), ESI(−)	8.51	1.52	0.13	1.41 × 10^−7^	146.04635	↓	Amino acid metabolism
L-Alanine	ESI(−)	6.60	1.45	0.15	6.07 × 10^−5^	88.04234	↓	Amino acid metabolism
Isocitrate	ESI(−)	11.22	1.24	0.20	2.12 × 10^−3^	191.01933	↓	TCA cycle
Hypoxanthine	ESI(+), ESI(−)	2.37	1.20	0.24	3.44 × 10^−4^	135.03135	↓	Purine metabolism
Xanthine	ESI(+)	3.44	1.13	0.32	1.10 × 10^−4^	153.03962	↓	Purine metabolism
PC(16:0/16:0)	ESI(+)	1.00	1.13	0.32	7.10 × 10^−5^	778.53738	↓	Lipid metabolism
L-Proline	ESI(+)	1.73	1.94	0.05	2.21 × 10^−3^	157.09589	↓	Amino acid metabolism
L-Phenylalanine	ESI(+)	5.50	2.13	0.04	4.55 × 10^−8^	165.07486	↓	Amino acid metabolism
L-Histidine	ESI(+)	13.58	1.40	0.17	1.42 × 10^−3^	156.07622	↓	Amino acid metabolism
L-Glutamine	ESI(+)	8.15	1.01	0.38	3.42 × 10^−4^	147.07577	↓	Amino acid metabolism
L-Citrulline	ESI(+)	7.54	1.82	0.08	6.17 × 10^−4^	217.12910	↓	Amino acid metabolism
L-Carnosine	ESI(+)	6.52	1.88	8.54	4.06 × 10^−5^	227.11334	↑	Amino acid metabolism
L-Arginine	ESI(+)	14.07	1.06	0.26	0.05	175.11853	↓	Amino acid metabolism
Imidazoleacetic acid	ESI(+)	7.40	1.47	3.44	1.61 × 10^−6^	270.11914	↑	Amino acid metabolism
GMP^172^	ESI(+)	10.42	1.04	0.37	1.09 × 10^−5^	364.06465	↓	Purine metabolism
Guanosine	ESI(+), ESI(−)	4.04	1.17	2.27	0.04	282.08322	↑	Purine metabolism
D-Mannitol	ESI(+)	5.49	2.00	0.06	2.54 × 10^−8^	183.08529	↓	Fructose and mannose metabolism
D-Glucose 6-phosphate	ESI(+), ESI(−)	11.68	1.55	0.16	1.50 × 10^−6^	278.06307	↓	Glycolysis/gluconeogenesis
D-Glucose	ESI(+)	11.68	1.57	0.15	4.24 × 10^−5^	243.02608	↓	Glycolysis/gluconeogenesis
Beta-D-Fructose 6-phosphate	ESI(+)	10.94	1.50	0.18	1.54 × 10^−5^	243.02559	↓	Fructose and mannose metabolism
Argininosuccinic acid	ESI(+), ESI(−)	11.46	1.38	0.19	1.63 × 10^−3^	289.11420	↓	Amino acid metabolism
alpha-Linolenic acid	ESI(+)	1.06	1.50	0.17	1.43 × 10^−4^	279.23093	↓	Lipid metabolism
AMP	ESI(+), ESI(−)	10.11	1.34	0.14	4.84 × 10^−3^	346.05357	↓	Purine metabolism
Deoxyadenosine	ESI(+)	1.97	1.06	2.29	0.06	252.10843	↑	Purine metabolism
Urocanic acid	ESI(+)	9.76	1.21	0.26	1.59 × 10^−3^	156.07635	↓	Amino acid metabolism
UMP	ESI(+), ESI(−)	10.45	1.57	0.15	6.45 × 10^−5^	325.04258	↓	Pyrimidine metabolism
UDP	ESI(+)	10.44	1.96	0.07	1.75 × 10^−7^	405.00844	↓	Pyrimidine metabolism
UDP-N-acetylglucosamine	ESI(+), ESI(−)	10.00	2.18	0.04	2.07 × 10^−8^	608.08737	↓	Peptidoglycan biosynthesis
UDP-D-Glucose	ESI(−)	10.42	1.90	0.06	4.60 × 10^−5^	565.04443	↓	Galactose metabolism
UDP-D-Galactose	ESI(+)	10.44	2.00	0.06	2.03 × 10^−7^	584.08782	↓	Galactose metabolism
Thiamine	ESI(+)	8.07	1.20	0.28	4.73 × 10^−4^	265.11128	↓	Vitamin metabolism
S-Methyl-5'-thioadenosine	ESI(+), ESI(−)	1.28	1.74	0.07	1.34 × 10^−6^	296.08064	↓	Purine metabolism
Sebacic acid	ESI(−)	5.92	1.59	0.10	3.22 × 10^−3^	201.11226	↓	Lipid metabolism
GMP^173^	ESI(+)	10.42	1.04	0.37	1.09 × 10^−5^	364.06465	↓	Purine metabolism
Sarcosine	ESI(+)	6.64	1.23	0.28	2.73 × 10^−5^	90.05550	↓	Amino acid metabolism
sn-Glycerol 3-phosphoethanolamine	ESI(−)	8.45	1.73	0.08	1.99 × 10^−5^	214.04807	↓	Lipid metabolism
Pantothenate	ESI(+)	3.96	2.21	0.03	9.13 × 10^−6^	220.11742	↓	Vitamin metabolism
Nicotinate	ESI(+), ESI(−)	2.79	2.27	0.03	5.47 × 10^−8^	124.03858	↓	Vitamin metabolism
ADMA	ESI(+)	13.49	1.21	0.29	0.02	203.14994	↓	Amino acid metabolism
Phenyllactic acid	ESI(−)	1.40	2.07	0.03	2.98 × 10^−4^	165.05476	↓	Others
N-Acetylputrescine	ESI(+)	6.97	1.05	0.21	0.03	131.11737	↓	Others
N-Acetyl-D-Glucosamine 6-Phosphate	ESI(+)	10.80	1.64	0.14	3.75 × 10^−6^	302.06304	↓	Others
N-Acetyl-D-glucosamine	ESI(+)	4.25	1.48	0.17	7.86 × 10^−5^	222.09609	↓	Others
N6,N6,N6-Trimethyl-L-lysine	ESI(+)	14.00	1.81	0.13	4.84 × 10^−5^	189.15917	↓	Amino acid metabolism
N,N′-Diacetylchitobiose	ESI(+)	6.80	1.84	0.09	2.35 × 10^−8^	425.17553	↓	Others
N-(omega)-Hydroxyarginine	ESI(+)	10.22	1.13	2.21	2.11 × 10^−3^	232.13999	↑	Amino acid metabolism
Maltotriose	ESI(+)	11.43	1.71	0.12	6.16 × 10^−6^	522.20183	↓	Others
L-Saccharopine	ESI(+), ESI(−)	10.75	1.80	0.10	3.11 × 10^−5^	277.13906	↓	Amino acid metabolism
O-Phosphotyrosine	ESI(−)	10.44	1.54	0.12	5.58 × 10^−5^	261.03667	↓	Others
Pipecolic acid	ESI(+)	5.37	1.53	0.13	0.01	130.08548	↓	Others
L-Methionine	ESI(+)	4.42	1.22	2.16	6.75 × 10^−6^	150.05752	↑	Amino acid metabolism
N-Acetylmannosamine	ESI(−)	4.75	1.43	0.12	5.70 × 10^−3^	220.08164	↓	Others
N-Acetylglucosamine 1-phosphate	ESI(−)	10.43	1.50	0.13	2.91 × 10^−4^	300.04740	↓	Others
Mesaconic acid	ESI(−)	10.27	1.09	0.28	1.63 × 10^−5^	129.01997	↓	Others
L-Pyroglutamic acid	ESI(+), ESI(−)	8.18	1.81	0.08	4.89 × 10^−5^	188.05548	↓	Amino acid metabolism
Homocitrate	ESI(−)	10.94	1.73	0.11	5.10 × 10^−5^	205.03501	↓	Amino acid metabolism
Levulinic acid	ESI(+)	8.79	1.84	0.09	6.42 × 10^−6^	134.08050	↓	Others
Isovalerylglycine	ESI(+)	9.47	1.62	0.14	4.42 × 10^−6^	177.12276	↓	Others
Isomaltose	ESI(+)	8.73	1.86	0.09	3.98 × 10^−8^	360.14947	↓	Starch and sucrose metabolism
Glycylproline	ESI(+)	9.37	2.02	0.06	1.89 × 10^−7^	233.11260	↓	Amino acid metabolism
Glycyl-L-leucine	ESI(+), ESI(−)	4.16	2.30	29.32	4.42 × 10^−4^	189.12269	↑	Amino acid metabolism
Glycerophosphocholine	ESI(+)	8.28	1.70	0.12	3.03 × 10^−10^	258.11003	↓	Lipid metabolism
Glutathione disulfide	ESI(+), ESI(−)	13.03	1.07	0.29	5.57 × 10^−3^	613.15817	↓	Glutathione metabolism
Glycerol 3-phosphate	ESI(+), ESI(−)	10.12	1.53	0.13	2.13 × 10^−5^	171.00594	↓	Lipid metabolism
L-Gamma-Glutamylcysteine	ESI(+)	9.39	1.65	0.12	1.61 × 10^−4^	292.10237	↓	Glutathione metabolism
D-Mannose-6-phosphate	ESI(+)	11.34	1.54	0.16	3.10 × 10^−6^	243.02578	↓	Fructose and mannose metabolism
D-Biotin	ESI(+)	3.92	1.39	0.22	3.70 × 10^−7^	245.09493	↓	Vitamin metabolism
D-Alanyl-D-alanine	ESI(+)	6.31	1.99	0.06	1.19 × 10^−5^	143.08073	↓	Amino acid metabolism
CMP	ESI(+), ESI(−)	11.16	1.17	0.26	3.54 × 10^−3^	324.05874	↓	Pyrimidine metabolism
CDP-choline	ESI(+), ESI(−)	10.48	1.42	0.19	6.84 × 10^−4^	489.11378	↓	Others
Fructose 1-phosphate	ESI(−)	11.67	1.39	0.18	1.10 × 10^−5^	259.02169	↓	Glycolysis/gluconeogenesis
D-Glucosamine 1-phosphate	ESI(−)	9.37	1.62	0.11	9.85 × 10^−6^	258.03715	↓	Others
Azelaic acid	ESI(−)	6.52	1.11	0.24	3.40 × 10^−3^	187.09704	↓	Others
Betaine	ESI(+)	4.43	1.61	0.15	5.08 × 10^−9^	118.08595	↓	Amino acid metabolism
Beta-D-Fructose 2-phosphate	ESI(+)	10.96	1.22	0.28	3.37 × 10^−5^	261.03660	↓	Fructose and mannose metabolism
Allocystathionine	ESI(+)	13.25	1.05	0.32	0.01	223.07394	↓	Others
alpha-ketoglutarate	ESI(−)	7.42	1.66	0.09	4.02 × 10^−3^	145.01419	↓	TCA cycle
2-Oxoadipic acid	ESI(−)	3.08	1.33	2.42	5.07 × 10^−4^	141.01637	↑	Amino acid metabolism
2-Ethyl-2-Hydroxybutyric acid	ESI(−)	1.57	2.08	0.03	6.94 × 10^−4^	131.07108	↓	Others
Acetylcarnitine	ESI(+)	5.30	2.24	0.03	2.36 × 10^−9^	204.12232	↓	Others
7-Methylguanosine	ESI(+)	5.82	1.29	0.08	0.10	298.11150	↓	Purine metabolism
4-Pyridoxic acid	ESI(+)	4.26	1.67	0.13	1.49 × 10^−7^	244.07854	↓	Vitamin metabolism
4-Guanidinobutyric acid	ESI(+)	6.99	1.11	0.30	6.75 × 10^−4^	146.09158	↓	Others
3-Hydroxyanthranilic acid	ESI(+)	6.58	1.93	0.07	2.75 × 10^−6^	154.04900	↓	Others
1-Methyladenosine	ESI(+)	4.87	1.01	0.36	5.55 × 10^−4^	282.11910	↓	Purine metabolism
(S)-2-Hydroxyglutarate	ESI(−)	8.34	1.77	0.07	1.14 × 10^−3^	147.02980	↓	Others
(S)-2-aminobutyric acid	ESI(−)	8.51	1.55	0.13	2.87 × 10^−7^	102.05681	↓	Others

*Trend: Relative metabolite levels in B. cinerea mycelia treated with TTO in comparison with control (↑, upregulated; ↓, downregulated). PC, phosphatidylcholine; AMP, adenosine monophosphate; GMP, guanosine 5′-monophosphate; UMP, uridine 5′-monophosphate; UDP, uridine 5′-diphosphate; ADMA, NG, NG-dimethyl-L-arginine; CMP, cytidine 5′-monophosphate. GMP^172^ and GMP^173^ designate different molecules; their adducts are (M+H)^+^ and (M+H-H_2_O)^+^, respectively. Differences with VIP > 1 and 0.05 < P < 0.1 are considered significant*.

### Hierarchical clustering analysis

**Figure 2:**
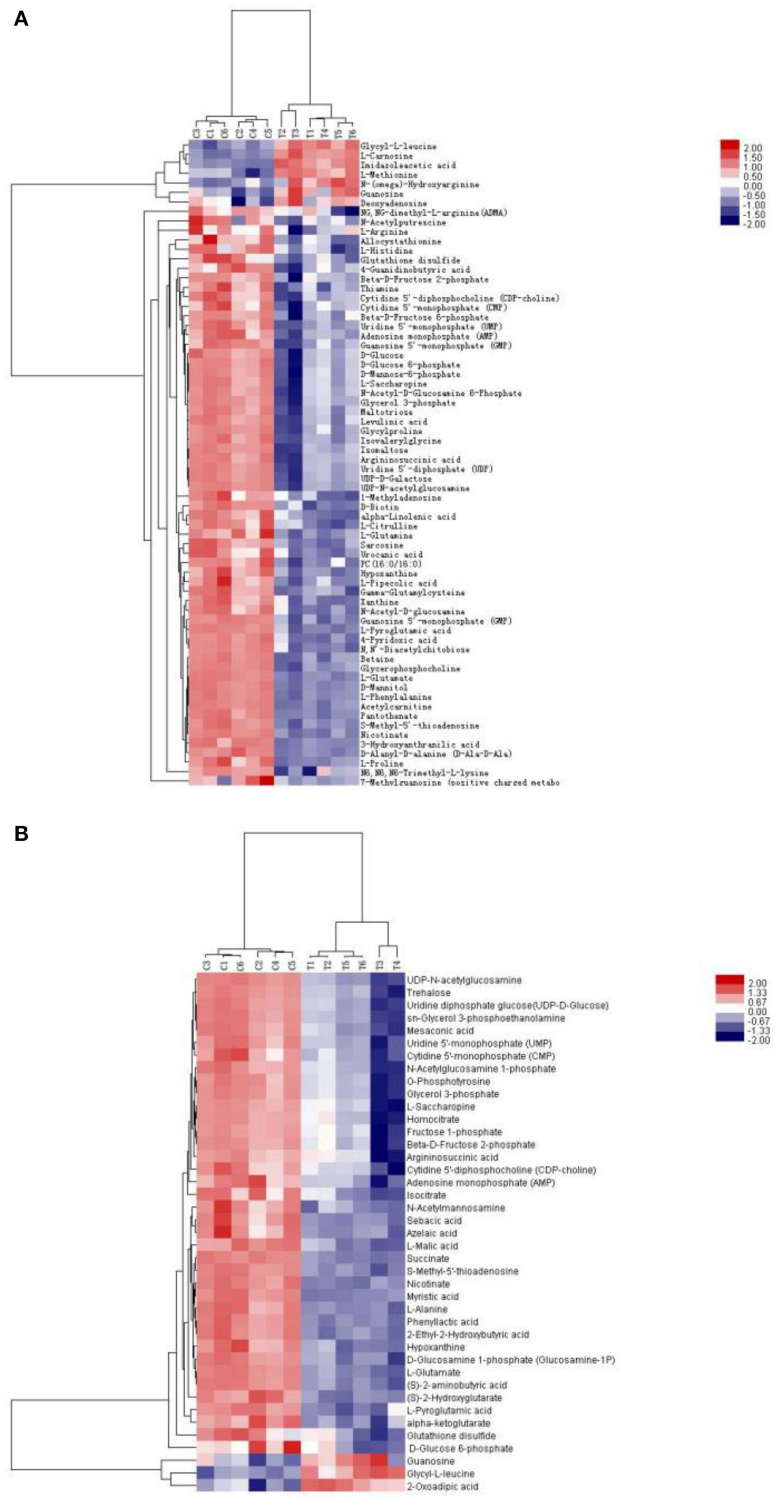
Heat map visualizing changes in levels of potential biomarkers in positive mode **(A)** and negative mode **(B)**. Rows: biomarkers; Columns: samples. The color key indicates the biomarker content ratio (blue: lowest; red: highest.) C, control sample; T, TTO-treated sample.

Table 1 shows the metabolic changes in fungal cells after treatment with TTO. The metabolites identified include products and intermediates generated by the TCA cycle, fatty acids, amino acids, and carbohydrates. The results suggest that *B. cinerea* adapts to environmental changes by regulating metabolic pathways. To provide an intuitive representation of the discriminatory power of the selected biomarkers in untreated and TTO-treated *B. cinerea*, a visual hierarchical clustering analysis (HCA, Figure 2) was performed. Each rectangle in the heat map represents one metabolite and is colored based on a normalized scale from −2 (low) to 2 (high). Differences between control and treated groups are the result of metabolite alterations after treatment with TTO. In positive mode (Figure 2A), seven metabolites [glycyl-L-leucine, L-carnosine, imidazoleacetic acid, L-methionine, N-(omega)-hydroxyarginine, guanosine, and deoxyadenosine] were upregulated in the TTO-treated sample, accounting for 10.3% of metabolites in positive mode. Other metabolites such as L-arginine, D-glucose 6-phosphate, phosphatidylcholine, alpha-linolenic acid, D-mannitol, and L-glutamate were downregulated in the TTO-treated sample, accounting for 89.7% of metabolites in positive mode. In negative mode (Figure 2B), glycyl-L-leucine, 2-oxoadipic acid, and guanosine were upregulated in the TTO-treated sample, accounting for 7.3% of metabolites in negative mode, while other metabolites such as isocitrate, L-malic acid, succinate, L-alanine, and trehalose were downregulated in the TTO-treated sample, accounting for 92.7% of metabolites in negative mode.

**Figure 3:**
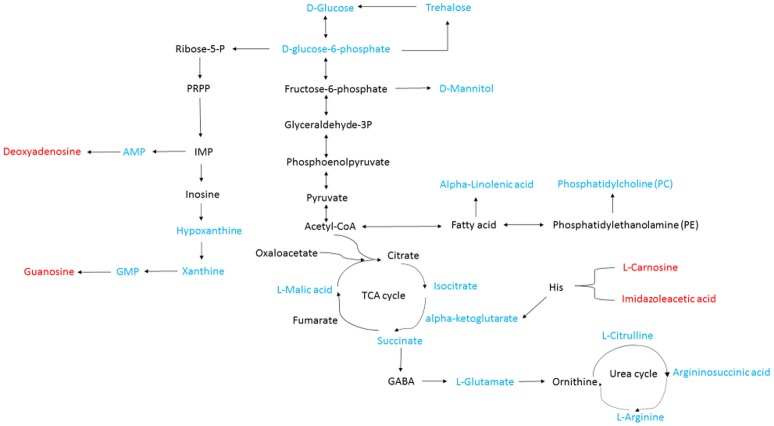
General biosynthetic pathway for selected metabolites as obtained from the KEGG database. Metabolites in red denote metabolites that are relatively more abundant in the TTO-treated sample, while metabolites in blue denote metabolites that are relatively less abundant.

Isocitrate, L-malic acid, succinate, and α-ketoglutarate are all involved in the TCA cycle. The contents of these compounds were all markedly decreased by 81.2, 84.1, 94.9, and 91.9%, respectively, in TTO-treated *B. cinerea* compared to the untreated control (Figure 2). Phosphatidylcholine and alpha-linolenic acid are related to the fatty acid. The levels of phosphatidylcholine and alpha-linolenic acid compared with control downregulated 68.3% and 83.3% respectively in TTO-treated cells (Figure 3). As illustrated in Table 1, the detected L-carnosine and imidazoleacetic acid in TTO-treated groups compared with control was distinctly increased to 8.54- and 3.44-fold, respectively.

### Effects of TTO on changes of key enzymes activities involved in TCA cycle, H_2_O_2_ accumulation, and leakage of cell membrane

As shown in Table 2, the activities of MDH, SDH, CS, ICDH and α-KGDH in untreated cells was 54.87 U/g, 23.75 U/g, 366.24 U/g, 647.50 U/g and 355.55 U/g, respectively. After treatment with TTO at 5 mL/L for 2 h, the activities for these enzymes decreased about 79.3, 78.9, 83.3, 66.0, and 91.7%, respectively. It indicated that all of these key enzymes involved in TCA cycle were significantly reduced in *B. cinerea* treated with TTO.

**Table 2:** Effect of TTO on SDH, MDH, CS, ICDH, α-KGDH, activities and H_2_O_2_ content in *B. cinerea* cells.

**Treatment**	SDH (U/g)^*^	MDH (U/g)^*^	CS (U/g)^*^	ICDH (U/g)^*^	α-KGDH (U/g)^*^	H_2_O_2_ (mmol/g prot)^*^
Control	23.75 ± 2.54^a^	54.87 ± 1.24^a^	366.24 ± 25.44^a^	647.50 ± 18.72^a^	355.55 ± 17.96^a^	43.65 ± 6.85^b^
TTO	5.00 ± 1.53^b^	11.35 ± 1.50^b^	61.16 ± 9.22^b^	220.29 ± 41.46^b^	29.46 ± 3.74^b^	120.95 ± 12.25^a^

a, b *differences are significant at P < 0.05 based on Duncan's multiple range tests*.

* *Values are presented as mean ± standard deviation*.

**Figure 4:**
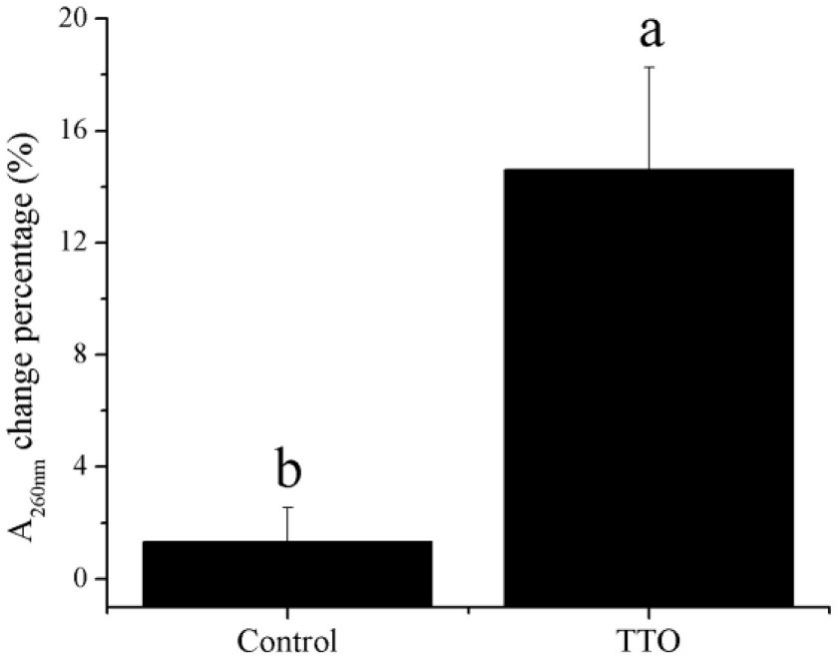
Effects of TTO treatment on *B. cinerea* as measured by percentage absorbance change at 260 nm. Vertical bars represent the standard deviation of the means. a,b: significant differences at *P* < 0.05 level.

Meanwhile, H_2_O_2_ content increased from 43.65 mmol/g prot to 120.95 mmol/g prot because of TTO treatment (Table 2). Absorbance at 260 nm increased by 14% after TTO treatment for 2 h, while the control group increased by only 1% (Figure 4). It was suggested that TTO led to the accumulation of H_2_O_2_ and the leakage of cell membrane.

## Discussion

Evidence suggests that essential oils damage fungal cell membranes, resulting in prolific leakage of ions, and nutrients. The leakage can be measured as an increase in absorbance at 260 nm and increasing conductivity in the medium (Hood et al., 2010; Tao et al., 2014). In our metabolomics analysis (Table 1), the majority of metabolites (83) were downregulated in the TTO-treated sample, perhaps due to the disruption of the cell membrane and metabolite leakage. But some metabolites (8) were upregulated in the TTO-treated sample. Shao et al. (2013a) showed that TTO treatment causes membrane leakage in *B. cinerea*, an increase in the activity of alkaline phosphatase, and an increase in palmitic acid (C16:0), stearic acid (C18:0), and oleic acid (C18:1) content was observed. Therefore, even after membrane integrity is compromised, some compounds may accumulate, possibly in response to stress.

The TCA cycle is the metabolic process by which ATP is generated in mitochondria, and is widely conserved in all oxidative organisms (Regev-Rudzki, 2005; Mailloux et al., 2007). The pathway contains several key enzymes (SDH, MDH, CS, ICDH, α-KGDH) and produces critical intermediates such as isocitrate, succinate, malate, and alpha-ketoglutarate (Li et al., 2015; Guo et al., 2016). As shown in Figure 2, TTO caused decreases in the levels of isocitrate, L-malic acid, succinate, and α-ketoglutarate. Mousavi et al. demonstrated that the levels of metabolites related to the TCA cycle, such as malic acid and glucose 6-phosphate, decreased in *E. coli* after treatment with cinnamaldehyde, suggesting downregulation of the TCA cycle (Mousavi et al., 2016). It is therefore possible that the TCA cycle of *B. cinerea* is also inhibited by TTO treatment.

To test our hypothesis, we measured the activities of MDH, SDH, CS, ICDH, and α-KGDH, key enzymes associated with the TCA cycle. As shown in Table 2, treatment with TTO at 5 mL/L for 2 h markedly decreased activities for these enzymes. In *Penicillium digitatum* cells, citral treatment inhibits the TCA pathway, as demonstrated by the reduction in citric acid content and reduction in the activities of CS, SDH, ICDH, and α-KGDH, although MDH activity increases (Zheng et al., 2015). However, MDH and SDH activities in *Sclerotinia sclerotiorum* and *Rhizopus nigricans* cells are decreased by dill seed essential oil and cinnamon oil, respectively (Li et al., 2014; Wang et al., 2015). Essential oils cause mitochondrial dysfunction by decreasing the activities of TCA-related enzymes, causing the accumulation of reactive oxygen species (ROS) and ultimately resulting in cell apoptosis (Tian et al., 2012; Zheng et al., 2015). Li et al. (2017) revealed that TTO severely damaged mitochondria of *B. cinerea*, resulting in matrix loss and increased mitochondrial irregularity. H_2_O_2_, an important ROS, can cause membrane lipid peroxidation and generation of protein carbonyl groups (Singh et al., 2015; Wang et al., 2016). TTO treatment increased H_2_O_2_ (Table 2), potentially resulting in oxidative stress and changes in the composition of cell membrane fatty acids. Together, these results suggest that TTO inhibits TCA cycle, possibly causing mitochondrial dysfunction and oxidation stress in *B. cinerea*.

Phosphatidylcholine is the major polar lipid in *B. cinerea*, while alpha-linolenate is the primary fatty acid (Griffiths et al., 2003). Both are major components of the cell membrane. Dill oil, which targets the plasma membrane of *A. flavus*, disrupts ergosterol biosynthesis (Khan et al., 2010; Tian et al., 2012). Citral exposure affects the expression of some genes involved in cell membrane-related pathways such as fatty acid biosynthesis and fatty acid metabolism, suggesting that it destroys cell membrane integrity (Ouyang et al., 2016). Citral, octanal, and α-terpineol decrease the total lipid content in *Geotrichum citri-aurantii* cells, causing the destruction of the cell membrane (Zhou et al., 2014). We previously reported that TTO treatment decreases the unsaturated/saturated fatty acid ratio in *B. cinerea*, suggesting that cell membrane disruption is related to changes in fatty acid composition (Shao et al., 2013a). In this study, phosphatidylcholine and alpha-linolenic acid were downregulated in TTO-treated cells (Figure 3), as well as the leakage of cell membrane (Figure 4). These results imply that TTO promotes membrane leakage either directly, or indirectly by interfering with membrane lipid biosynthesis and fatty acid composition.

A dramatic increase in H_2_O_2_ content was detected in *B. cinerea* cells incubated with TTO (Table 2), which demonstrates that TTO treatment increases the level of free radicals, resulting in oxidative stress. L-carnosine (beta-alanyl-L-histidine) is a natural dipeptide with antioxidant activity and functions as a scavenger for free-radicals (Hipkiss, 2009; Boldyrev et al., 2013). L-carnosine levels increased in the TTO-treated sample, perhaps as a protective response against H_2_O_2_-induced oxidative stress. L-carnosine has a similar function in red blood cells subjected to oxidative stress by H_2_O_2_ (Aydogan et al., 2008).

The binding of γ-aminobutyric acid (GABA) to the GABA receptor triggers changes in plasma membrane ionic permeability (Perfilova and Tiurenkov, 2011). At least three types of GABA receptors, designated GABA_*A*_, GABA_*B*_, and GABA_*C*_, have been characterized (Qian and Dowling, 1993). Imidazoleacetic acid is a major catabolite of histamine and functions as a GABA_*C*_ receptor antagonist (Liu et al., 2006). Our data show that TTO treatment increases membrane permeability in *B. cinerea* (Figure 4) and also increases the level of imidazoleacetic acid in *B. cinerea* (Figure 3). The increase in the level of imidazoleacetic acid may inhibit binding to the GABA_C_ receptor, conferring partial protection against membrane leakage induced by TTO-treatment.

## Conclusion

This report presents the first metabolomics analysis of *B. cinerea* after treatment by TTO, and demonstrates that TTO disrupts the TCA cycle, affects the level of various cellular components, and causes cell membrane leakage. The damage results in mitochondrial dysfunction and oxidative stress. L-carnosine and imidazoleacetic acid levels increase in response to these events.

## Author contributions

JX and XS designed the experiments. JX, YL, and YW performed the experiments. FX and HW analyzed the data. JX, XS, and HW drafted the manuscript. All authors read and approved the final manuscript.

### Conflict of interest statement

The authors declare that the research was conducted in the absence of any commercial or financial relationships that could be construed as a potential conflict of interest.
